# The COVID-19 pandemic and the worldwide online interest in telepsychiatry: an infodemiological study from 2004 to 2022

**DOI:** 10.3389/fdgth.2025.1425684

**Published:** 2025-04-01

**Authors:** Rowalt Alibudbud

**Affiliations:** Department of Sociology and Behavioral Sciences, De La Salle University, Manila City, Philippines

**Keywords:** telepsychiatry, internet-based interventions, infodemiology, Google, Wikipedia, online mental health services, digital mental health

## Abstract

**Introduction:**

Studies call for the further assessment and understanding of public interests and concerns about telepsychiatry, especially during the COVID-19 pandemic. Since telepsychiatry services are accessed through the Internet, this study analyzed online searches and queries to determine telepsychiatry-related interests and concerns over time. The findings can inform the development and customization of online telepsychiatry resources and services, enabling a more effective response to public needs.

**Materials and methods:**

This study determined public concerns and interests in telepsychiatry using data from Google Trends and Wikipedia from 2004 to 2022. These platforms were selected for their large global market share. After describing the data, bootstrap for independent sample tests of search volumes and Wikipedia page views before and during the COVID-19 pandemic.

**Results:**

The highest interest in telepsychiatry was observed in high-income countries. Search volumes for telepsychiatry increased, while Wikipedia page views decreased during the COVID-19 pandemic. The top and rising queries that can be incorporated into telepsychiatry websites include telepsychiatry concepts, jobs, services, costs, and locations.

**Discussion:**

The findings support that the use of the Internet for telepsychiatry information increased compared to previous years, especially during the start of the COVID-19 pandemic. There may also be a higher interest in telepsychiatry among high-income nations compared to low and middle-income countries. Furthermore, the study also supports that digital information should be tailored to respond to public needs and expectations by incorporating telepsychiatry-related concepts, jobs, services, costs, and locations.

## 1 Introduction

Digital technology, including telemedicine and telepsychiatry, has the potential to transform clinical practice (1). Telemedicine has a long history and was first described in 1879 as the long-distance transmission of medical appointments and knowledge using the telephone (2). On the other hand, telepsychiatry is a specific application of telemedicine within psychiatry. It was documented in the 1950s when the Nebraska Psychiatric Institute utilized videoconferencing for educational and consultative activities (2). With telepsychiatry, psychiatric services can have greater reach and lower costs (2, 3).

As the COVID-19 pandemic ensued in 2020, demands for telepsychiatry services have risen (4). Research about telepsychiatry has revealed several of its weaknesses, including its limited application in low- and middle-income countries (LMIC) (5, 6) and in areas with low internet penetration (i.e., rural areas) (7). On the other hand, its advantages have also been documented, including its similar effectiveness to clinical practice (5), lower cost (4), relative high acceptability among clinicians (1, 6), its potential for improving patient outcomes (1) and it's potential to increase access to mental healthcare in low-resource settings (6). However, there have also been calls for a comprehensive evaluation of the utilization of telepsychiatry as demand continues to grow, emphasizing the need to assess its acceptability, limitations, and effectiveness across different service providers and users (4, 8). Consequently, further research is needed to gain a comprehensive understanding of the gaps in public needs, concerns, interests, and expectations regarding telepsychiatry. Such efforts can provide significant insights into public perceptions and help improve its adoption. The findings could also inform the development and customization of online telepsychiatry information and services, ensuring they better address public needs and concerns. In recent years, public needs, concerns, interests, and expectations about telepsychiatry can be studied using infodemiological techniques and big data analysis.

Since telehealth services, including telepsychiatry, can be accessed through the Internet aside from traditional telephone communications (2, 3, 7), the number of Internet searches and specific queries can be analyzed to determine online interest and concerns over time. The analysis of internet data to inform health policy is subsumed under the field of infodemiology (9). Infodemiology has been used to analyze public concerns and interest in various health phenomena, including communicable and non-communicable diseases (10–13).

One of the most important tools in infodemiology is Google Trends (10, 14). The increasing utility of Google Trends can be attributed to the rising global internet use and access, as well as the predominant global market share of Google among search engines (10, 12, 14, 15). Furthermore, data from Google Trends can be complemented with other online data, including Wikipedia page views, to further comprehend online information and interest (11, 12, 15, 16). This is because of the high utility and web traffic of Wikipedia as a source of health information compared to institutional health websites (i.e., the National Health Service, National Institute of Health, and the World Health Organization) (12, 17). Furthermore, both data from Google Trends and Wikipedia have also been analyzed to determine public interest in various psychiatric and mental health phenomena, including subdisciplines, services, concepts, and various disorders (i.e., Dementia, Substance Use Disorder) (11–16). Therefore, data from Google Trends and Wikipedia can be analyzed to understand public concerns and interest in telepsychiatry.

### 1.1 Objectives and significance

Overall, previous studies call for the further assessment to gain a comprehensive understanding of the gaps in public interests and concerns about telepsychiatry, especially during the COVID-19 pandemic. In response, the present study explored public concerns and interests in telepsychiatry using an infodemiological approach, leveraging data from Google Trends and Wikipedia both before and during the COVID-19 pandemic. First, it analyzed relative search volumes (RSV) and Wikipedia page views to assess public interest in and utilization of telepsychiatry-related information on these platforms. Second, it compared RSV data across countries to identify those with the highest levels of interest in telepsychiatry. Third, it examined changes in RSV and Wikipedia page views before and during the pandemic to evaluate the pandemic's impact on online information use. Finally, it analyzed search-related queries to uncover public concerns about telepsychiatry. By doing so, the study can contribute to a better understanding of public interests and concerns about telepsychiatry and help improve its utilization. Its findings can also be utilized in creating and tailoring online telepsychiatry information and services to better respond to public interests and concerns.

## 2 Materials and methods

### 2.1 Data sources and measures

Google trends provides RSV and top and rising search-related queries and topics from January 2004 onwards (10, 14, 18). Google automatically computes RSV by anonymizing its users’ information, categorizing their search queries, and aggregating them into topics. This algorithm results in a relative number that is scaled from 0 to 100 (10, 14, 18). In this scale, 0 and 100 represent the lowest and highest search volumes in the specified period and location (10, 14, 18). In addition, Google Trends also provides top and rising search-related queries and topics, which are the queries and topics with the most growth compared to the previous period that users also searched for concerning a keyword of interest (10, 14, 18). Similar to previous studies (10–12, 15, 16), the present study utilized RSV and top and rising search-related queries to understand public concerns and interests about telepsychiatry.

The Wikimedia website provides data about Wikipedia page views from July 2015 onwards (12). Wikipedia page views can also be used to analyze the web traffic regarding a particular Wikipedia website. Similar to previous studies (12, 15, 16), the present study utilized page views to understand the utilization of Wikipedia information about telepsychiatry.

### 2.2 Data collection

The present study followed the methodological framework suggested by Mavragani (19) and Alibudbud (14) to gather monthly RSV for web searches in all categories. Data from Google Trends were collected using the keyword “Telepsychiatry (Topic)” with a time filter from January 2004 to December 2022 and a location filter of “worldwide” (accessed on April 13, 2023; available from trends.google.com). The keyword “Telepsychiatry (Topic)” was chosen because a “Topic” keyword represents a collection of terms that convey the same concept across different languages (10). After, the RSV and top and rising search-related queries and country rankings were downloaded using this keyword and filters.

Wikipedia page views were gathered following the methodology of Mondia et al. (12) from the Wikimedia website. First, the Wikipedia page for telepsychiatry was selected by inputting “Telepsychiatry” in the Page feature (accessed on April 13, 2023; available from trends.google.com). Then, before downloading the data, the date type was adjusted to “Monthly,” the date setting was set “from July 2015 to December 2022”, and the agent filter was changed to “user” only. This agent filter was selected to avoid web crawlers and limit the data to people who view the Wikipedia page for telepsychiatry.

No human participant was involved in this study, and the data was publicly available. Thus, the present study may not necessitate ethical approval.

### 2.3 Data analysis

After describing the Wikipedia page views, RSV, country rankings, and top and rising search-related topics and queries, the bootstrap methodology in the infodemiological study of Mavragani and Gkillas (20) was performed to determine any significant difference in the RSV and page views before and during the COVID-19 pandemic. A bootstrap technique, involving repeated sampling with replacement, was used to estimate sampling distributions and address challenges posed by limited sample sizes and potential small-sample estimation bias (20, 21). A bootstrap sample size of 999 iterations was chosen, exceeding the minimum recommended size of 399 by Davidson and MacKinnon (21) to limit power loss to less than 1% based on their simulations. This method was applied to compare mean differences in RSV and Wikipedia page views before and during the COVID-19 pandemic. Independent sample tests with bootstrapping were conducted using the Statistical Package for the Social Sciences (SPSS) to determine statistical significance. For the present study, RSV were grouped and marked according to two periods which include the period before the pandemic (before March 2020), and when COVID-19 was pronounced a pandemic (March 2020 onwards) by the WHO (22). A *p*-value of <.05 was considered significant.

## 3 Results

### 3.1 Trends of search volumes and Wikipedia page views for telepsychiatry

**Figure 1:**
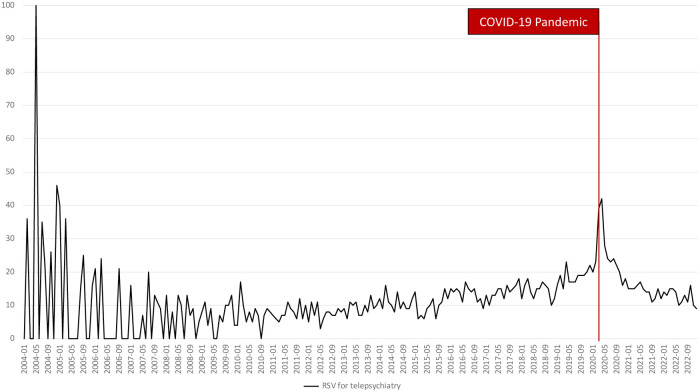
Global trends of RSV for telepsychiatry from 2004 to 2022 with a line indicating the COVID-19 pandemic.

**Figure 2:**
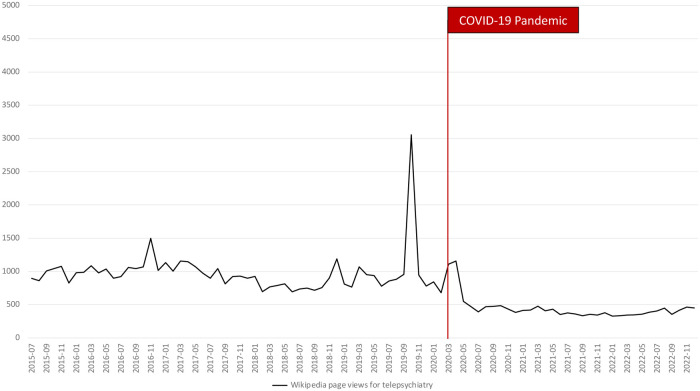
Global trends of Wikipedia page views for telepsychiatry from 2015 to 2022 with a line indicating the COVID-19 pandemic.

Figure 1 shows that the observed search volumes from Google Trends have an increasing trend where it started with an RSV of 0 and ended at 9, representing a 900% increase over the study period. As the COVID-19 pandemic began, it was also observed that RSV rose to 39 before declining to 9 by December 2022. In addition, Figure 2 shows that Wikipedia page views started at 896 and ended at 450, representing almost a 100% increase over the study period. Like the RSV, it also rose during the start of the pandemic at 1,108. These findings suggest that the use of Google and Wikipedia as a source of information about telepsychiatry increased during the study period. Likewise, a sudden rise in their use was also observed during the pandemic's start.

### 3.2 Mean differences and bootstrap for independent sample tests of RSV and Wikipedia page views before and during the COVID-19 pandemic

The mean differences between RSV and page views before and during the COVID-19 pandemic. The RSV before the pandemic (mean = 10.825, SD = 10.105) was significantly lower than the RSV during the pandemic (mean = 17.000, SD = 7.422). On the other hand, the Wikipedia page views before the pandemic (mean = 969.929, SD = 321.128) were higher than the page views during the pandemic (mean = 447.206, SD = 182.157). The differences in RSV and Wikipedia page views were significant, *p* < .05.

These findings suggest that the COVID-19 pandemic has influenced the public interest and use of Google and Wikipedia information for telepsychiatry. Public utilization and interest in Google information for telepsychiatry were significantly higher during the pandemic, while the use of Wikipedia information decreased during the pandemic.

### 3.3 Public interest in telepsychiatry among different countries

Google Trends ranked 15 countries based on their RSV for telepsychiatry (see Table 1). The United States was the highest-ranked country, followed by Australia, Canada, the United Kingdom, and the Philippines. The countries not ranked by Google Trends have low search volumes for telepsychiatry to be listed. These findings suggest that the highest public interest in telepsychiatry came from predominantly high-income countries.

**Table 1:** Country rankings based on search volumes for telepsychiatry.

Country	RSV
United States	100
Australia	43
Canada	43
United Kingdom	20
Philippines	20
Romania	18
India	16
Sweden	13
South Korea	13
Thailand	9
Vietnam	8
Germany	8
Indonesia	7
Saudi Arabia	6
France	5
Brazil	2

### 3.4 Related public concerns for telepsychiatry

Table 2 shows the types of top and rising search-related topics and queries for telepsychiatry. These search-related topics and queries revolved around telepsychiatry-related concepts, jobs, services, cost, and location. These findings suggest that people are interested and searching the Internet for telepsychiatry-related concepts, jobs, services, costs, and locations.

**Table 2:** Types of search-related topics and queries for telepsychiatry.

Types	Associated terms	Examples of queries and topics
Concepts	Searches about terms related to telepsychiatry concepts	“telepsychiatry” “what is telepsychiatry” “telemedicine”
Jobs	Searches about telepsychiatry jobs, employment, and other related opportunities	“telepsychiatry jobs” “psychiatry jobs” “Job” “Salary”
Services	Searches about various telepsychiatry services, types of services, and service providers	“telepsychiatry companies” “InSight Telepsychiatry, LLC” “telepsychiatry nurse practitioner jobs” “psychiatrist” “Therapy” “New York”
Cost	Searches about the costs of telepsychiatry	“telepsychiatry cost”
Location	Searches about various general and specific locations	“telepsychiatry near me” “New York” “Arizona”

## 4 Discussion

The findings suggest an increased search for online information about telepsychiatry in Google and Wikipedia between 2004 and 2022. This finding is consistent with previous studies revealing that the Internet is increasingly utilized as a source of health information (10, 11, 14, 15). The findings from this study extends this notion of increase use of online health information to telepsychiatry concepts in the internet. This higher internet use may be explained by the rising internet penetration across the globe (10, 11).

The present study also observed a sudden rise in the use of Google and Wikipedia for telepsychiatry information as the COVID-19 pandemic started. As the pandemic ensued, it also found that the public utilization and interest in Google information for telepsychiatry further increased compared to pre-pandemic levels. On the other hand, it was also observed that the use of Wikipedia information during the pandemic was lower than pre-pandemic levels. The increased utilization of Google for information may be attributed to the heightened demand for telepsychiatry services during the pandemic (4). While Wikipedia's health-related content is widely read (17), the present study indicates a decline in readership specifically interested in telepsychiatry during this period. This decline could be linked to the growing reliance on other reputable health websites, such as those of the World Health Organization and the National Institutes of Health (17). However, further research is needed to investigate the rise in Google searches alongside the decrease in Wikipedia page views about telepsychiatry during the pandemic.

A digital divide consistent with socioeconomic disparities was also found in this study, where the highest public interest in telepsychiatry came from predominantly high-income countries. This higher interest among high-income nations is consistent with previous notions that digital technology innovations, such as telepsychiatry, might have limited application in LMIC and other low-resource settings (5, 6). This higher use of digital information from the internet has previously been attributed to the greater accessibility of the internet and its applications in high-income countries compared to LMIC (5, 6, 10, 12, 14, 15). Nonetheless, as internet penetration and utilization around the world improves (10, 12, 14, 15), there is a need to tailor telepsychiatry information so that it can respond better to public concerns and interests. The findings suggest that the top and rising queries of Google users about telepsychiatry are telepsychiatry-related concepts, jobs, services, costs, and locations. Therefore, internet information about telepsychiatry can better respond to the growing digital and public needs by including telepsychiatry concepts, job postings, and services, including their locations, reach, and costs.

### 4.1 Limitations

While the present study highlighted several insights regarding telepsychiatry, its findings must be viewed in light of its limitations. First, the study utilized data from Wikipedia and Google. Although both platforms are widely used globally, certain areas, such as China, may primarily rely on other web search engines like Baidu. Future research could therefore explore data from these alternative search engines to assess the utilization of telepsychiatry information on their platforms. Second, Google Trends data may have limited application in areas with low freedom of speech and internet access. Therefore, other research designs (i.e., surveys and interviews) can be done in these regions. Third, search volumes are a surrogate measure of public interests and concerns. Hence, traditional measures, such as population-based surveys, should be utilized to further and comprehensively understand public interest and concerns about telepsychiatry.

## 5 Conclusion

Overall, previous studies observed the increasing use of the Internet as a source of health information. The present study supports that this increasing internet use for health information also extends to telepsychiatry. Moreover, the use of the Internet for telepsychiatry information increased compared to previous years, especially during the start of the COVID-19 pandemic. There may also be a higher interest in telepsychiatry among high-income nations compared to LMIC. Therefore, digital information should be tailored to respond to public needs and expectations by incorporating telepsychiatry-related concepts, jobs, services, costs, and locations.

## Data Availability

The raw data supporting the conclusions of this article will be made available on request to the corresponding author/s.
